# Effect of different mycobionts on symbiotic germination and seedling growth of *Dendrobium officinale*, an important medicinal orchid

**DOI:** 10.1186/s40529-019-0278-6

**Published:** 2020-01-27

**Authors:** Ying Zhang, Yuan-Yuan Li, Xiao-Mei Chen, Shun-Xing Guo, Yung-I Lee

**Affiliations:** 1 Institute of Medicinal Plant Development, Chinese Academy of Medical Sciences & Peking Union Medical College, Beijing, 100193 People’s Republic of China; 2 Biology Department, National Museum of Natural Science, Taichung, 40453 Taiwan; 3 Department of Life Sciences, National Chung Hsing University, Taichung, 40227 Taiwan

**Keywords:** *Dendrobium*, Mycorrhiza, Seed germination, Seedling growth, Crude polysaccharides

## Abstract

**Background:**

Orchids maintain a symbiotic relationship with mycorrhizal fungi in the lifecycle. Previous reports indicated that diverse mycobionts may have different roles during orchid growth and development. Although various mycorrhizal fungi have been isolated from *Dendrobium* roots and protocorms, little is known about their specific effects on seed germination and seedling growth. To understand the specific role of isolated fungal strains (i.e., *Tulasnella* and *Sebacina*), we used symbiotic culture to compare the effect of 6 fungal strains on seed germination and seedling growth of *Dendrobium officinale*, an important Chinese medicinal orchid.

**Results:**

In symbiotic germination tests, 6 fungal strains (4 *Tulasnella* strains and 2 *Sebacina* strains) promoted seed germination with different efficiencies. Seeds inoculated with *Tulasnella* strains S6 and S7 conferred higher germination percentage and faster protocorm development than other fungal strains. In symbiotic cultures, seedlings inoculated with *Sebacina* strain S3 had optimal fresh and dry matter yield. Also, *Tulasnella* strains S6 and S7 promoted seedling growth with good fresh and dry matter yield. *Sebacina* strain S2 inoculation greatly enhanced root and tiller production and the content of total crude polysaccharides, although seedlings were smaller with less fresh and dry matter yield than other seedlings.

**Conclusions:**

*Tulasnella* and *Sebacina* strains could promote seed germination and seedling growth of *D. officinale* with different efficiencies. Our results suggest a non-specific mycorrhizal association and development-dependent preference. Our data provide the basic knowledge for use of different fungal strains in conservation and/or production practices of *D. officinale*.

## Background

A common example of widespread mutualism is the association of mycorrhiza and land plants, whereby fungi retrieve mineral nutrition in the soil and pass it back to plants and plants contribute the products of photosynthesis to fungi (Smith and Read 2008). In orchids, a compatible mycorrhizal association is a requirement for seed germination under natural conditions (Rasmussen 1995). During seed germination and early growth of protocorms, mineral nutrients are transferred from fungi to the orchid, a phenomenon known as “mycoheterotrophy” (Leake 1994). After successful infection, fungal hyphae coil and form pelotons within the cortical cells (Smith and Read 2008). Afterward, the pelotons senesce and collapse to release nutrients in orchid cells (Kuga et al. 2014). The formation and degradation of pelotons play a key role in the exchange and absorption of nutrients between an orchid and its mycorrhizal fungus (Dearnaley and Cameron 2017; Fochi et al. 2017).

Most green orchids form mycorrhizae with polyphyletic Rhizoctonia-like fungi, including *Tulasnella*, *Ceratobasidium*, *Thanatephorus* and *Sebacina* clade B (Dearnaley et al. 2012). Orchids may have high specificity in their fungal partners; an example is mycoheterotrophic orchids (Leake 1994; Bidartondo 2005) such as *Corallorhiza striata* complex (Barrett et al. 2010) and *Hexalectris* (Kennedy et al. 2011). In contrast, other orchids may associate with diverse sets of fungal partners; for example, *Cypripedium californicum* associates with Tulasnellaceae, Ceratobasidiaceae, and Sebacinales (Shefferson et al. 2007). Moreover, Tulasnellaceae, Thelephoraceae, Ceratobasidiaceae, Sebacinales, Russulaceae and Clavulinaceae were detected in *Cymbidium goeringii* and *Cymbidium lancifolium* (Ogura-Tsujita et al. 2012). Also, a succession of fungal colonization over the orchid life cycle occurs; for example, *Gastrodia elata*, a mycoheterotrophic orchid, needs *Mycena* for seed germination, but subsequent colonization by *Armillaria* is required for the orchid’s further development (Xu and Mu 1990). Although orchids are colonized by different fungi, not all colonized fungi have the same effect on the growth and development of orchids. In *Vanilla*, different mycorrhizal fungal isolates from roots had dissimilar effects on growth and survival (Porras-Alfaro and Bayman, 2007). In *Dendrobium*, *Tulasnella* isolated from *D. aphyllum* protocorms could stimulate germination, but *Trichoderma* could not improve germination (Zi et al. 2014).

*Dendrobium officinale*, one of the most important *Dendrobium* species in China, has long been used in traditional Chinese medicine to treat chronic diseases (Pharmacopoeia Committee of the P. R. China 2005). Previous investigations showed various fungal mycobionts identified in and/or isolated from roots or protocorms of medicinal *Dendrobium* species (Chen et al. 2012; Tan et al. 2014; Zi et al. 2014; Wang et al. 2017), which suggests no high specificity in the fungal association. However, the roles of various fungal strains isolated from *Dendrobium* species have never been tested systematically with controlled in vitro culture methods.

To understand the effects of various fungal strains on ontogenetic stages in orchid, we compared the effect of 6 fungal strains of *Tulasnella* and *Sebacina* on symbiotic germination and tested their ability to promote seedling growth by examining the growth rate and crude polysaccharides content. Knowledge of mycorrhizal association in medicinal *Dendrobium* species would be helpful for propagation, commercial cultivation and conservation.

## Methods

### Plant materials

Plants of *D. officinale* were maintained in a plantation in Jinhua, Zhejiang, China. At the time of anthesis, in March, flowers were hand-pollinated, and mature capsules were collected just before dehiscence, in November, for the following experiments. In total, 10 capsules were collected for experiments.

### Fungal strains for symbiotic cultures

In our laboratory, we have developed efficient methods for fungal isolation from *D. officinale* protocorms and roots (Li et al. 2018a). In this study, *Sebacina* strains S2 and S3 were isolated from mycorrhizal protocorms by the technique of in situ seed baiting in the natural habitat in Yunnan Province of Southwestern China, and *Tulasnella* strains S4, S5, S6 and S7 were isolated from roots of mature plants in the same area. These fungal strains were maintained in our laboratory and deposited at the microbiological center of the Institute of Medicinal Plant Development, Chinese Academy of Medical Sciences & Peking Union Medical College, Beijing (see Additional file 1: Table S1). Before symbiotic culture, fungal strains were inoculated on potato dextrose agar medium (potato 200 g L^−1^, glucose 20 g L^−1^, agar 12 g L^−1^, pH 5.2 before autoclaving) according to the procedure by Li et al. (2018b) in darkness at 25 ± 1 °C for 7 days. The actively growing mycelia from the colony margin were used as the fungal inoculum in subsequent symbiotic cultures.

### Molecular identification and phylogenetic analysis

DNA was extracted from pure mycelium cultures of fungi by using the Rapid Plant Genomic DNA Isolation Kit (Sangon Biotech, Shanghai) according to the manufacturer’s instructions. The internal transcribed spacer (ITS) region of the fungal nuclear rRNA gene was amplified with the primer pairs ITS1F/ITS4R (ITS1F: 5′-TCCGTAGGTGAACCTGCGG-3′; ITS1R: 5′-TCCTCCGCTTATTGATATGC-3′) (White et al. 1990; Gardes and Bruns 1993). The PCR amplification was initial denaturing at 95 °C for 5 min, followed by 35 cycles of denaturing at 95 °C for 1 min each, annealing at 55 °C for 1 min, extension at 72 °C for 1 min, and final extension for 5 min at 72 °C. Sequences were identified by a BLAST search against the NCBI sequence database (GenBank). For phylogenetic analysis, other ITS sequences of *Tulasnella* and *Sebacina* from various orchids were obtained from GenBank. The sequence of *Armillaria sinapina* was used as the outgroup. DNA sequences were aligned by using the ClustalW algorithm in MEGA 7.01, followed by manual adjustment. Distance trees were obtained by using the neighbor-joining (NJ) method (Saitou and Nei 1987) with a Tajima-Nei method. For assessing relative robustness for branches, the bootstrap method was used with 1000 replicates (Felsenstein 1985).

### Symbiotic germination

Before sowing seeds, oat meal agar medium (OMA: oat 4 g L^−1^, agar 8 g L^−1^, pH 5.2 before autoclaving) placed in a 9-cm diameter Petri dish was inoculated with 4 pieces of fungal inoculum for each fungal strain, then Petri dishes were placed in darkness at 25 ± 1 °C for 7 days as described by Li et al. (2018a, b). Capsules were rinsed under tap water for 10 min and surface-sterilized with 75% ethanol for 60 s, followed by immersion in 2.5% sodium hypochlorite solution for 15 min. After rinsing 3 times with sterile water, capsules were cut and opened to remove seeds. About 200 seeds were sown onto the surface of OMA medium in each Petri dish. 12 replicates (Petri dishes) were used for each fungal treatment. Petri dishes without fungal inoculum were the control. After inoculation, the exact number of seeds of each dish was counted under a stereomicroscope. The cultures were placed in a growth room under a 12/12-h light/dark photoperiod at 30 μmol m^−2^ s^−1^ (daylight fluorescent tubes FL-20D/18, 20W) at 25 ± 1 °C.

### Symbiotic culture of seedlings

To compare the effect of different fungal strains on seedling growth in symbiotic culture, young seedlings were obtained from asymbiotic culture before inoculation. Mature seeds were removed from capsules and placed onto modified Murashige and Skoog medium (Murashige and Skoog 1962), containing half-strength macroelements with full-strength microelements, vitamins, and amino acids and supplemented with 20 g L^−1^ potato homogenate, and 20 g L^−1^ sucrose, and solidified with 7 g L^−1^ agar. The pH of the medium was adjusted to 5.7 with 1 N NaOH solution before autoclaving at 101.33 kPa and 121 °C for 20 min. The cultures were placed in the growth room under a 12/12-h photoperiod at 25 ± 1 °C as described previously. After 6 months of sowing, seedlings about 3 cm tall were selected for symbiotic culture.

For symbiotic culture of seedlings, OMA medium combined with H1 basal salts (200 mg L^−1^ Ca(NO_3_)_2_·4H_2_O, 100 mg L^−1^ KCl, 200 mg L^−1^ KH_2_PO_4_, 100 mg L^−1^ MgSO_4_·7H_2_O, 100 mg L^−1^ yeast extract, 2 g L^−1^ sucrose) described by Rasmussen (1995) was used. Each glass bottle (9 cm diameter, 12.5 cm tall) contained 125 mL culture medium, and pH was adjusted to 5.7 before autoclaving. Before symbiotic cultures of seedlings, each glass bottle was inoculated with 4 pieces (0.5 cm^3^) of fungal inoculum. As the hyphae had spread over the surface of culture medium, uniform seedlings (450 mg fresh weight, single shoot of 3 cm tall with 5 roots) from asymbiotic cultures were selected and transferred to glass bottles; 12 replicates (glass bottles) were used, and each glass bottle contained 6 seedlings for each fungal treatment. After 3 months of symbiotic culture, the fresh weight, dry weight, shoot number and root number of each seedling were recorded. Glass bottles without fungal inoculum were the control. Symbiotic cultures were placed in the growth room under a 12/12-h photoperiod at 25 ± 1 °C as described previously.

### Histological and histochemical observations

Developing mycorrhizal protocorms were fixed in 1% glutaraldehyde in 0.1 M phosphate buffer (pH 6.8) for 4 h at room temperature and dehydrated with an ethanol series, then embedded in Technovit 7100 resin (Kulzer and Co., Wertheim, Germany) according to Yeung and Chan (2015). Serial, 3-μm-thick sections were cut and stained with Periodic acid–Schiff reaction for total insoluble carbohydrates, then counterstained with 0.05% (w/v) toluidine blue O (TBO) for general histology. Sections were observed under a light microscope (Axio ImagerA1, Carl Zeiss AG) and the images were captured digitally by using a CCD camera. For fungal hyphae staining, mycorrhizal protocorms were fixed as described above. Subsequently, samples were washed with 1× PBS (pH 7.4) for 3 times for 10 min, then incubated at room temperature for 90 min in 1× PBS containing the chitin-specific dye WGA-FITC at 10 μg L^−1^ (Molecular Probes, Karlsruhe, Germany). After a washing with 1x PBS for 3 times for 10 min, samples were mounted on glass slides and observed under a confocal microscope (LSM510, Carl Zeiss, Germany) with a 488-nm laser line and detected at 505–540 nm.

### Measurement of polysaccharides content

Polysaccharides were extracted as described (Wang et al. 2018) with minor modification. Seedling stems were dried in an oven at 80 °C for 24 h. Dried stems were ground into a fine powder and sieved through a 40-mesh sieve. Each ground sample of 20 mg was placed into a 2-mL centrifuge tube with 440 μL distilled water for about 2 h, then extraction was performed at 63 °C for 18 min in a KH5200DE ultrasonic instrument (Kunshan Hechuang Ultrasonic Machinery Co., Jiangsu, China). The extraction process was repeated 3 times, and supernatants were combined. The water extract was precipitated with 4volumes of absolute ethanol, kept at 4 °C for 24 h, then centrifuged at 5180×*g* at 4 °C for 20 min. The precipitate was precipitated in 80% ethanol, then dissolved in 1 mL water. After mixing, 100 μL polysaccharide aqueous solution and 1.9 mL water were added into a plugged test tube, then 1 mL of 5% phenol solution and 5 mL concentrated sulfuric acid were successively added to the same test tube. The test tube was placed into a boiling water bath for 20 min. After cooling, colorimetric determination was measured at 490 nm by using the EnSpire^®^ Multimode Plate Reader (PerkinElmer, USA). Standard glucose at 99.9% (CAS: 50-99-7) was purchased from the National Institutes for Food and Drug Control (Beijing), with glucose solutions (0, 12.5, 25, 50, 100 and 150 μg mL^−1^) as standards. The linear regression equation was y = 6.9023x + 0.0824 (R^2^ = 0.9993). The content of polysaccharides in each sample (Y) was calculated as follows:

$$ {\text{Y}} = \frac{{{\text{Cs }} \times {\text{V }} \times {\text{D}}}}{\text{M}} \times 100 $$

where Y is the content of crude polysaccharides (mg/100 mg), Cs is the concentration of glucose in the tested sample solution (μg mL^−1^), V is volume of the tested sample solution (mL), D is the dilution multiple of sample solution, and M is the sample weight (mg).

### Experimental design and data analysis

In symbiotic germination experiments, each Petri dish was observed and recorded under a stereomicroscope every 2 weeks after sowing. Germination and developmental stages of protocorms were defined and scored according to Stewart et al. (2003). Germination was defined as emergence of the embryo from the seed coat. All experiments (i.e., symbiotic cultures of seed germination and seedlings) were arranged in a completely randomized design. The data were analyzed by using one-way analysis of variance (ANOVA). Mean separation was performed by Fisher’s protected least significant difference test (*P* < 0.05) using SPSS 22.0 (IBM, Chicago, IL, USA).

## Results

### The effect of fungal strains on symbiotic germination

**Fig. 1:**
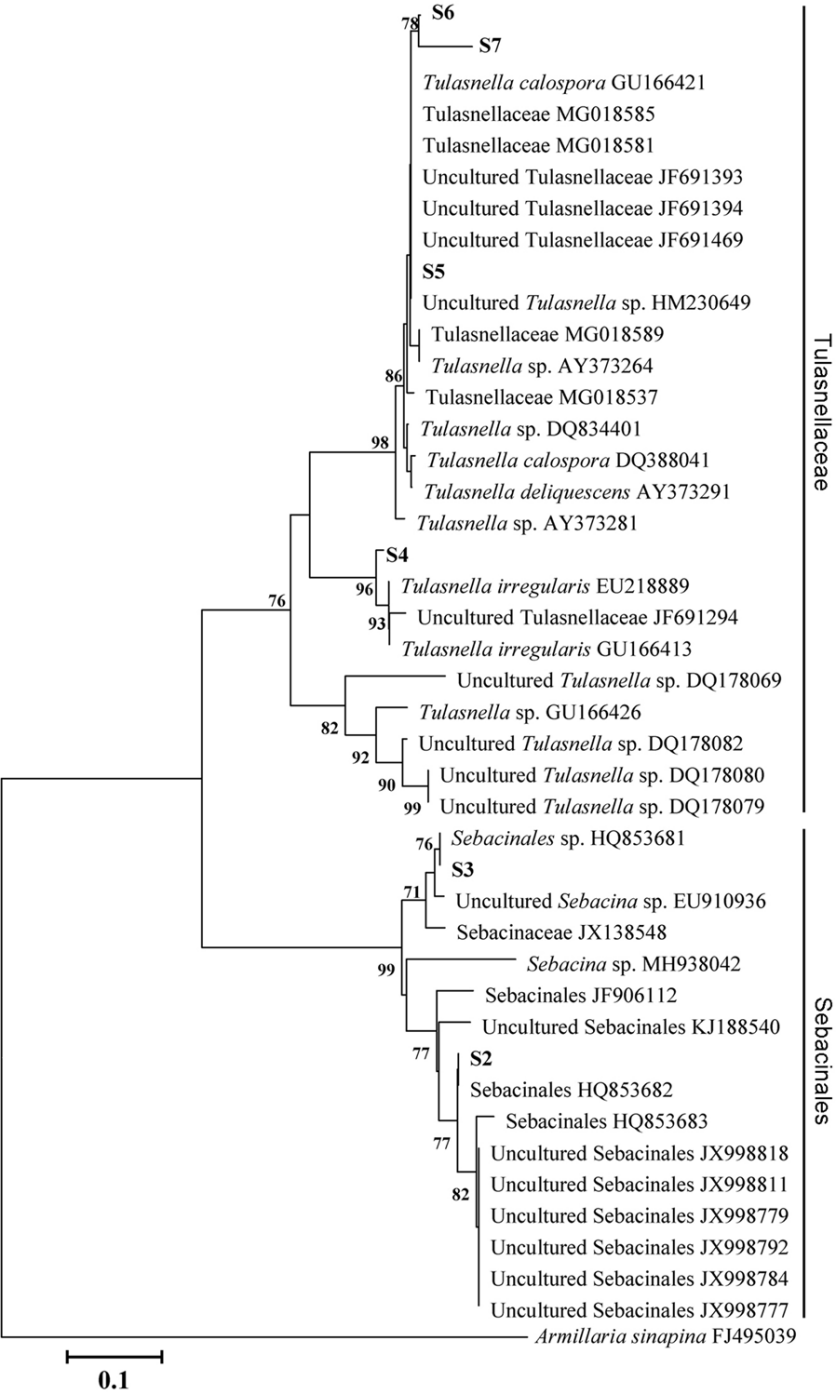
Phylogenetic relationships of the fungal strains used in this study based on neighbor-joining analysis of internal transcribed spacer rDNA sequences of Tulasnellaceae and Sebacinales available in GenBank. Bootstrap values (calculated from 1000 re-samplings) > 70% are shown at branches. *Armillaria sinapina* was the outgroup

The results of phylogenetic analysis indicated that 6 isolated Rhizoctonia-like fungal strains were clustered with Tulasnellaceae and Sebacinales (Fig. 1, Additional file 1: Table S1). According to molecular and morphological data, 4 *Tulasnella* and 2 *Sebacina* strains were identified. The cultures of *Tulasnella* strains S4, S5, S6 and S7 showed creamy white colonies, whereas those of *Sebacina* strains S2 and S3 exhibited yellowish white colonies. With the fungal strains S3, S6 and S7, growth was rapid on potato dextrose agar medium at 25 ± 1 °C, but with strains S2, S4 and S5, growth was slower (Additional file 2: Figure S1).

**Fig. 2:**
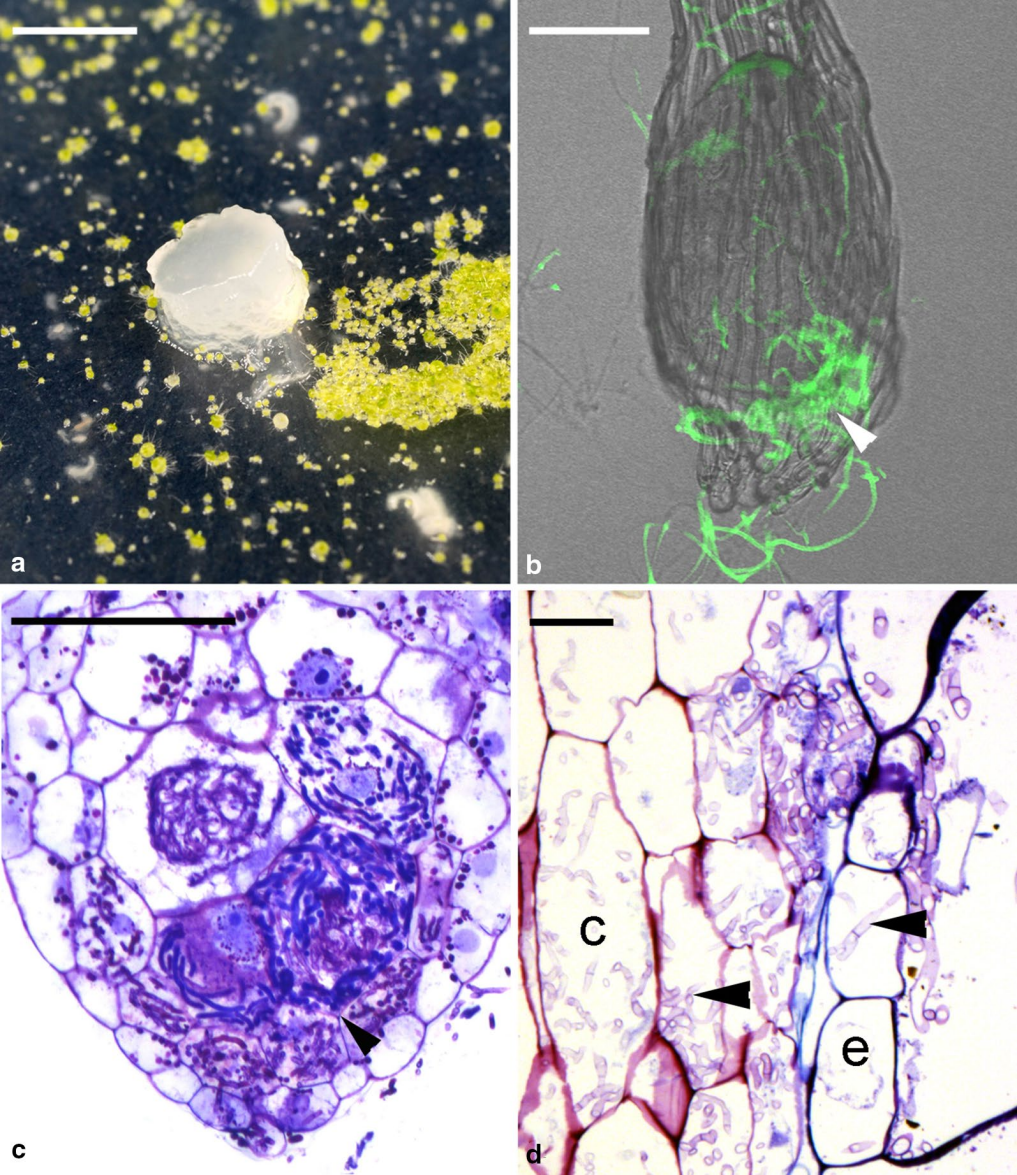
Symbiotic cultures of *D. officinale* seeds and seedlings. **a** Symbiotic seed germination of *D. officinale* seeds. Protocorms ruptured the seed coats and turned green. Scale bar = 10 mm. **b** Fungal staining using WGA-FITC (green) showing fungal hyphae (arrowhead) aggregated and penetrating the suspensor end of embryo. Bar = 40 µm. **c** Longitudinal section of a developing protocorm showing fungal hyphae (arrowhead) colonizing the basal cortical cells of an enlarged embryo. Scale bar = 50 µm. **d** Longitudinal section of a seedling root showing fungal hyphae (arrowheads) colonizing the epidermis (e) and cortical cells (c). Scale bar = 15 µm

In the symbiotic germination experiments, the 4 *Tulasnella* and 2 *Sebacina* strains could induce seed germination (Fig. 2a). After 1 week of inoculation, embryos had become swollen, and fungal hyphae congregated at the suspensor end of embryos (Fig. 2b). At this stage, minor seed coat rupture was observed. After 2 weeks of inoculation, embryos continued to enlarge, which resulted in a major rupture of the seed coat (Fig. 2c). Fungal hyphae had colonized primarily in the outer and inner cells at the basal part (the suspensor end) of protocorms and formed the intracellular pelotons (Fig. 2c).

**Fig. 3:**
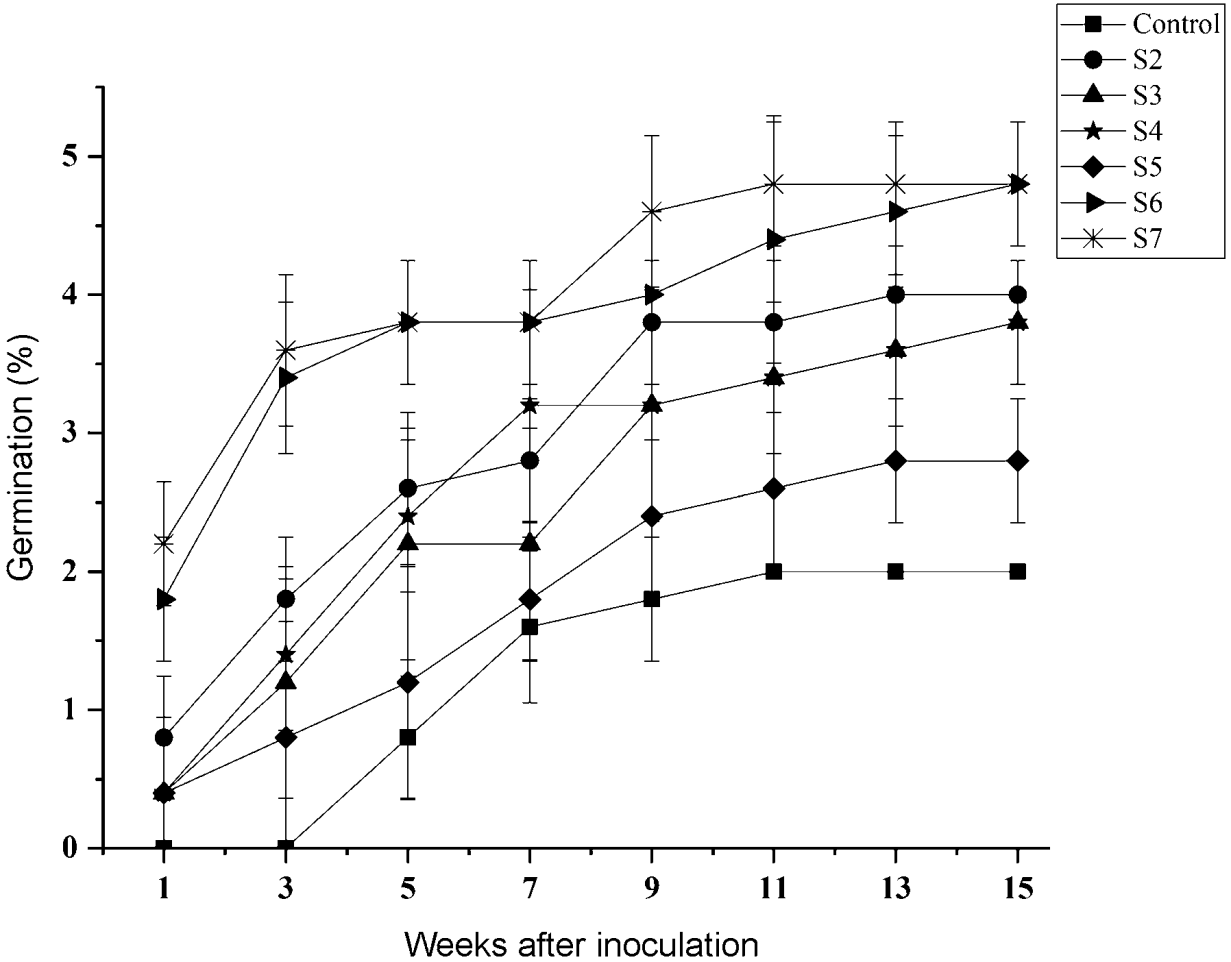
Germination rate of *D. officinale* seeds inoculated with different fungal strains. After 1 week of inoculation, germination was recorded for each treatment every 2 weeks. Data are mean ± SE (n = 3)

The 6 fungal strains had the potential to promote seed germination at different efficiencies (Fig. 3) and to different developing protocorm stages (Table 1, Additional file 3: Figure S2). In the control (asymbiotic OMA), seeds became swollen, with rupture of the seed coat (stage 2), but no further embryo development occurred. After 5 weeks of inoculation, seeds inoculated with *Tulasnella* strains S6 and S7 showed higher germination rate than the other fungal strains (Table 1). However, by 9 weeks of inoculation, seeds inoculated with S7 had more protocorms reach stage 5 than those inoculated with S6 (Table 1). Seeds inoculated with *Sebacina* strains S2 and S3 and *Tulasnella* strain S4 showed a similar germination rate. In contrast, seeds inoculated with *Tulasnella* strain S5 had a slower germination rate than the other fungal strains, and the development of protocorms reached only stage 3 by 15 weeks of inoculation.

**Table 1:** Effect of fungal strains on seed germination and protocorm development of *D. officinale* at 15 weeks of inoculation

Treatment	Ratio of seed germination and protocorm development (%)^b^						Total germination (%)^c^
	Stage 0	Stage 1	Stage 2	Stage 3	Stage 4	Stage 5	
Control^a^	25.72 ± 2.85^b^	60.04 ± 4.34^a^	14.24 ± 3.07^d^	0.00 ± 0.00^d^	0.00 ± 0.00^b^	0.00 ± 0.00^b^	14.24 ± 3.07^d^
S2	8.77 ± 1.30^c^	6.56 ± 3.95^bc^	25.97 ± 5.41^c^	50.23 ± 8.08^a^	8.48 ± 4.00^b^	0.00 ± 0.00^b^	84.67 ± 8.85^a^
S3	41.82 ± 2.63^a^	9.23 ± 0.67^bc^	14.86 ± 0.90^d^	28.94 ± 3.11^b^	5.14 ± 2.16^b^	0.00 ± 0.00^b^	48.95 ± 4.02^c^
S4	12.87 ± 10.01^c^	12.92 ± 5.41^b^	40.86 ± 8.43^b^	28.53 ± 2.59^bc^	4.69 ± 0.83^b^	0.00 ± 0.00^b^	74.08 ± 7.31^b^
S5	9.15 ± 1.95^c^	8.01 ± 1.24^bc^	64.20 ± 2.73^a^	18.80 ± 1.39^c^	0.00 ± 0.00^b^	0.00 ± 0.00^b^	83.00 ± 1.39^ab^
S6	5.47 ± 1.96^c^	10.40 ± 5.98^b^	37.43 ± 7.03^b^	23.60 ± 8.49^bc^	19.97 ± 6.49^a^	1.35 ± 1.09^b^	86.35 ± 3.07^a^
S7	5.10 ± 2.60^c^	1.83 ± 2.28^c^	7.08 ± 6.58^d^	7.84 ± 3.56^d^	19.20 ± 9.80^a^	59.17 ± 8.78^a^	93.29 ± 4.20^a^

^a^Control, the seeds without fungal inoculation

^b^Germination percentages (mean ± SE, n = 3) within columns marked by different letters are significantly different at *P* < 0.05 (Fisher’s protected least significant difference test)

^c^Germination was defined as emergence of the embryo from the seed coat (stage 2)

### The effect of fungal strains on seedling growth in symbiotic cultures

**Fig. 4:**
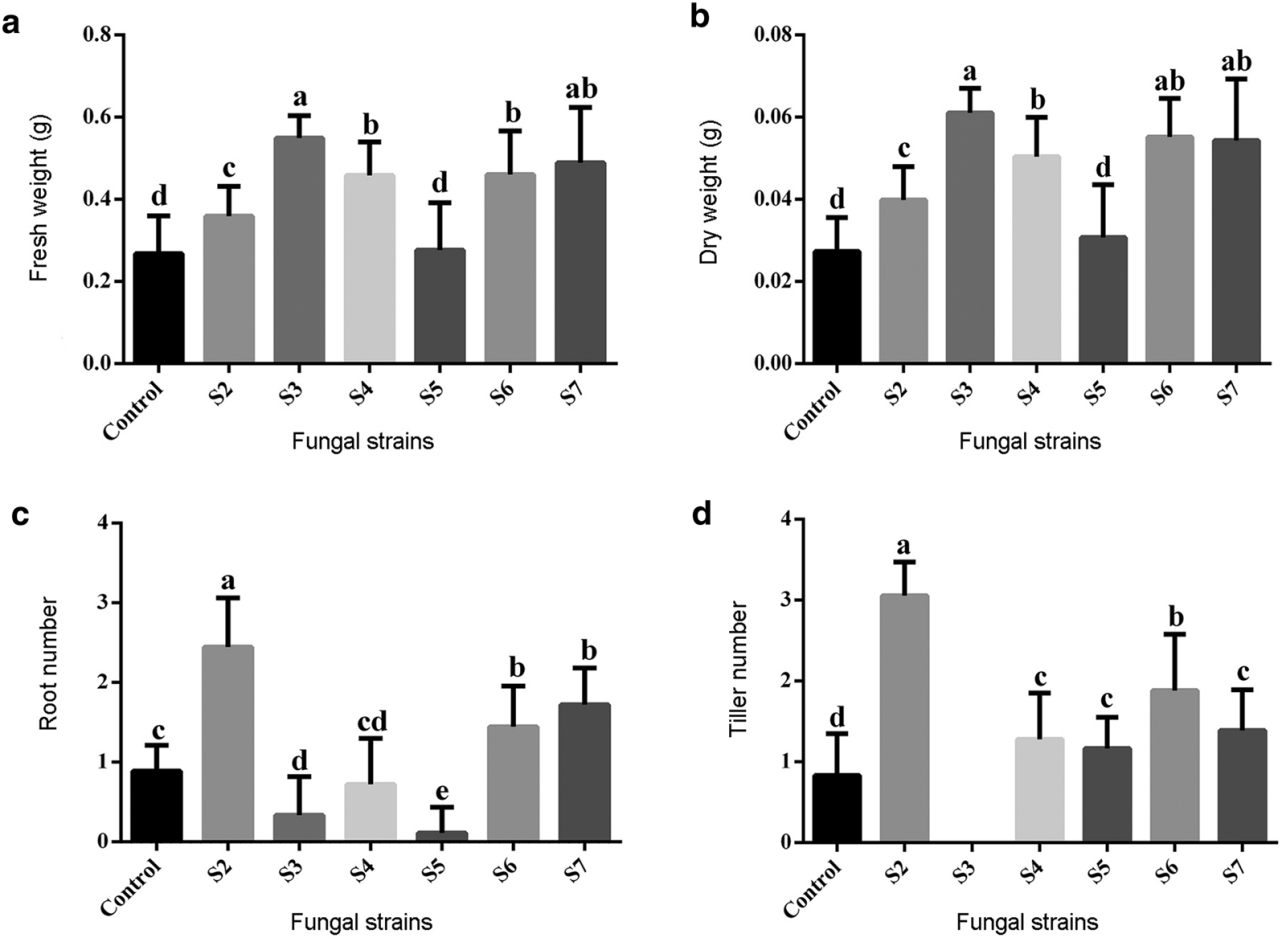
Effect of fungal strains on seedling growth of *D. officinale* at 15 weeks after inoculation. Change in **a** fresh weight, **b** dry weight, **c** root number, and **d** tiller number. Data are mean ± SE. Different letters above bars are significantly different at *P* < 0.05 (Fisher’s protected least significant difference test)

Light microscopy revealed that all fungal strains formed a symbiotic association with *D. officinale* seedlings, as evidenced by the presence of pelotons in the cortical region of roots (Fig. 2d). Growth of *D. officinale* seedlings differed greatly with the fungal inoculations (Fig. 4). Seedlings inoculated with *Sebacina* strain S3 showed optimal fresh and dry matter yield. Also, seedlings inoculated with *Tulasnella* strains S6 or S7 showed good fresh and dry matter yield (Fig. 4). Number of roots and tillers produced was significantly greater with *Sebacina* strain S2 inoculation, although the seedlings were smaller and accumulated relatively little fresh and dry matter (Fig. 4).

### The content of crude polysaccharides in stems

**Fig. 5:**
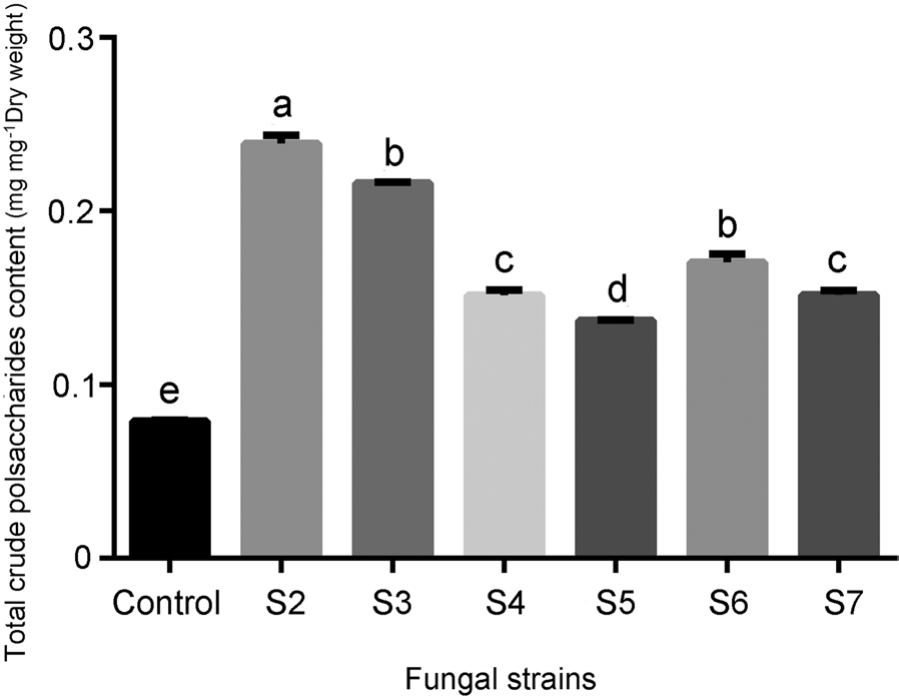
Effect of fungal strains on total crude polysaccharides content in stems of *D. officinale* at 15 weeks after inoculation. Data are mean ± SE. Different letters above bars are significantly different at *P* < 0.05 (Fisher’s protected least significant difference test)

Polysaccharides accumulation in stems was higher in all mycorrhizal seedlings than in the control without fungal inoculation (Fig. 5). Among the fungal strains tested, inoculation with the *Sebacina* strain S2 conferred the highest polysaccharides content.

## Discussion

Most green orchids are known to associate with fungi from the polyphyletic rhizoctonia group, with its different clades such as Serendipitaceae (Sebacinales), Tulasnellaceae and Ceratobasidiaceae (Batty et al. 2006; Valadares et al. 2011). The mycorrhizal association of orchids could be compatible with several fungal groups or be highly specific for a narrow group of fungi (Otero et al. 2002; Ma et al. 2003; McCormick et al. 2004; Shefferson et al. 2005; Suárez et al. 2006). Fungus isolated from a peloton in adult orchid roots and its role as a seed germination symbiont cannot been ascertained until the results of symbiotic germination are known (Hoang et al. 2017). Furthermore, the non-compatible fungi may stimulate orchid seed germination, but they could not support subsequent seedling development (Rasmussen et al. 2015). In *Dendrobium* species, various fungal symbionts such as Cantharellaceae, Sebacinales and Tulasnellaceae are present in the protocorm and roots of adult plants (Chen et al. 2012). In the report by Tan et al. (2014), the ITS sequence of an efficient *Tulasnella* strain JC-02 in promoting seed germination of *D. officinale* is identical to *Tulasnella* strains S6 in this study, suggesting this efficient fungal strain may be dominant in wild *Dendrobium* populations. In previous reports, *Sebacina* strains could stimulate seed germination and further protocorm development of *D. nobile* and *D. officinale* (Wang et al. 2011; Zhao et al. 2013). In this study, the results of symbiotic germination revealed that *D. officinale* is compatible with different *Tulasnella* and *Sebacina* fungal strains for seed germination (Table 1; Fig. 3), although the efficiency of the strains differs. While *Tulasnella* strains S4 and S5 are not close related to *Tulasnella* strains S6 and S7 (Fig. 1), they could stimulate seed germination and protocorm formation to stage 2 or 3 after 15 weeks of inoculation. Although they are not as effective as *Tulasnella* strains S6 and S7, a few seedlings were observed by 30 weeks of inoculation (data not shown). Our data suggest a non-specific fungal association (at least two different fungal clades, i.e. Sebacinales and Tulasnellaceae, Fig. 1) during germination in *D. officinale*.

In the present study, the mycobionts strains tested had different effects on growth and development of seedlings (Fig. 4). Although *Sebacina* strain S3 had little effect on seed germination, it significantly improved the growth of seedlings (i.e., optimal fresh and dry matter yield) (Figs. 4a, b). In orchids, mycorrhizal symbionts may switch in different developmental stages (McCormick et al. 2004; Rafter et al. 2016). Previous studies have demonstrated that compatible fungi for promoting seed germination may not be able to support subsequent seedling development (Bidartondo and Read 2008; Huynh et al. 2009; Rasmussen et al. 2015). Inoculation with *Sebacina* strain S2 greatly improved the number of roots and tillers of seedlings (Fig. 4c, d) but had little effect on yield of fresh and dry matter (Fig. 4a, b). The changes in plant growth pattern (i.e., dwarfism, multiple shoots and roots) after inoculation with *Sebacina* strain S2 may be attributed to hormonal compounds derived from mycorrhizal fungus. The production of plant hormones by symbiotic fungi can affect the growth and development of host plants (Hirsch et al. 1997). Further research into plant hormones produced by mycorrhizal fungi would provide insights into the growth and development of *Dendrobium* seedlings in symbiotic cultures.

Both *Sebacina* strains S2 and S3 were unable to support post-germination development beyond stage 3 by 15 weeks of inoculation (Table 1). In adult orchids, different fungal strains could be isolated from the same single peloton or from the same single root (Kristiansen et al. 2001; Raleigh et al. 2001; McKendrick et al. 2002; Taylor et al. 2003; Bougoure et al. 2005). Under natural conditions, the broadening and/or switch of a mycorrhizal association may enable orchids to adapt to the varied physiological changes during seedling development, including the switch to partial or full autotrophy, an increase in transpiration, or environmental fluctuations (Těšitelová et al. 2012).

The crude polysaccharides, the non-starch, hetero-polysaccharides, have been considered the main indicative ingredients in medicinal *Dendrobium* species (Meng et al. 2013; Dave and Shah 2015). Our data show a significant effect on the accumulation of crude polysaccharides in mycorrhizal seedlings of *D. officinale* as compared with the control (Fig. 5). Increased crude polysaccharides content has been reported in seedlings of *D. nobile* after fungal inoculation (Li et al. 2017). Moreover, we found a diverse effect on the growth and crude polysaccharides content of seedlings with different fungal treatments. Although conferring less fresh and dry matter yield, inoculation with *Sebacina* strain S2 resulted in the highest crude polysaccharides content as compared with other *Sebacina* and *Tulasnella* strains. Seedlings inoculated with *Sebacina* strain S3 had optimal fresh and dry matter yield and also relatively high crude polysaccharides content (about 21.6% less than S2). Fungal elicitors were found to affect the accumulation of active ingredients in medical plants by changing the expression of specific genes involved in secondary metabolite biosynthesis (Zhai et al. 2017).

*Tulasnella* strains S6 or S7 could stimulate seed germination and also support the growth of seedlings (Fig. 4a, b). For *Drakaea*, a single mycorrhizal symbiont could support growth from the protocorm to seedling and adult stages (Phillips et al. 2011). In the conservation or production practices of *D. officinale*, inoculation with *Tulasnella* strains S6 or S7 for all developing stages may simplify the operation. In terms of medicinal plant production, the addition of *Sebacina* strains S2 or S3 to young seedlings may increase crude polysaccharides content or fresh and dry matter yield.

## Conclusions

The present study demonstrates that for symbiotic germination*, D. officinale* is compatible with *Tulasnella* and *Sebacina* strains, thereby suggesting a non-specific mycorrhizal association. Seedlings inoculated with *Sebacina* strains S2 and S3 showed optimal crude polysaccharides content and fresh and dry matter yield, respectively, although with little effect on seed germination. These fungal strains tested in symbiotic cultures have different effects on growth and development of seedlings, which suggests development-dependent specificity. Our data provide basic knowledge of use of different fungal strains in conservation and/or production practices.

## Supplementary information

**Additional file 1: Table S1.** Fungal strains used in this study.

**Additional file 2: Figure S1.** Characteristics of mycobionts at 7 days with culture on potato dextrose agar medium. (A) fungal strain S2; (B) fungal strain S3; (C) fungal strain S4; (D) fungal strain S5; (E) fungal strain S6; (F) fungal strain S7. Scale bar = 1 cm.

**Additional file 3: Figure S2.** Developing stages of *D. officinale* from seed germination to protocorm formation. (A) Stage 0, embryos enclosed by intact seed coats. Scale bar = 0.5 mm. (B) Stage 1, swollen seeds after 1 week of inoculation. Scale bar = 0.5 mm. (C) Stage 2, swollen embryo rupturing the seed coat. Scale bar = 0.5 mm. (D) Stage3, green protocorm with shoot tip and rhizoids. Scale bar = 0.5 mm. (E) Stage 4, emergence of first leaf. Scale bar = 0.5 mm. (F) Stage 5, emergence of second leaf. Scale bar = 0.5 mm.

## Data Availability

Not applicable.
