# Supplementary material for: Effect of different mycobionts on symbiotic germination and seedling growth of Dendrobium officinale, an important medicinal orchid
Source: Bot Stud. 2020 Jan 27;61:2. doi: 10.1186/s40529-019-0278-6 (PMC6985412; doi:10.1186/s40529-019-0278-6)
Supplement: Supplementary file 1 — Additional file 1: Table S1. Fungal strains used in this study. [file 40529_2019_278_MOESM1_ESM.docx]

Table S1. The fungal strains used in this study.

| Strain | Sources | Taxon | GenBank accession |
| --- | --- | --- | --- |
| S2 | Protocorms | *Sebacina* sp. | HQ853682.1 |
| S3 | Protocorms | *Sebacina* sp. | HQ853681.1 |
| S4 | Adult roots | *Tulasnella* sp. | KX587486.1 |
| S5 | Adult roots | *Tulasnella* sp. | MN503661.1 |
| S6 | Adult roots | *Tulasnella* sp. | JN863900.1 |
| S7 | Adult roots | *Tulasnella* sp. | JN863901.1 |
