# Supplementary figures and images for: Effect of different mycobionts on symbiotic germination and seedling growth of Dendrobium officinale, an important medicinal orchid
Source: Bot Stud. 2020 Jan 27;61:2. doi: 10.1186/s40529-019-0278-6 (PMC6985412; doi:10.1186/s40529-019-0278-6)

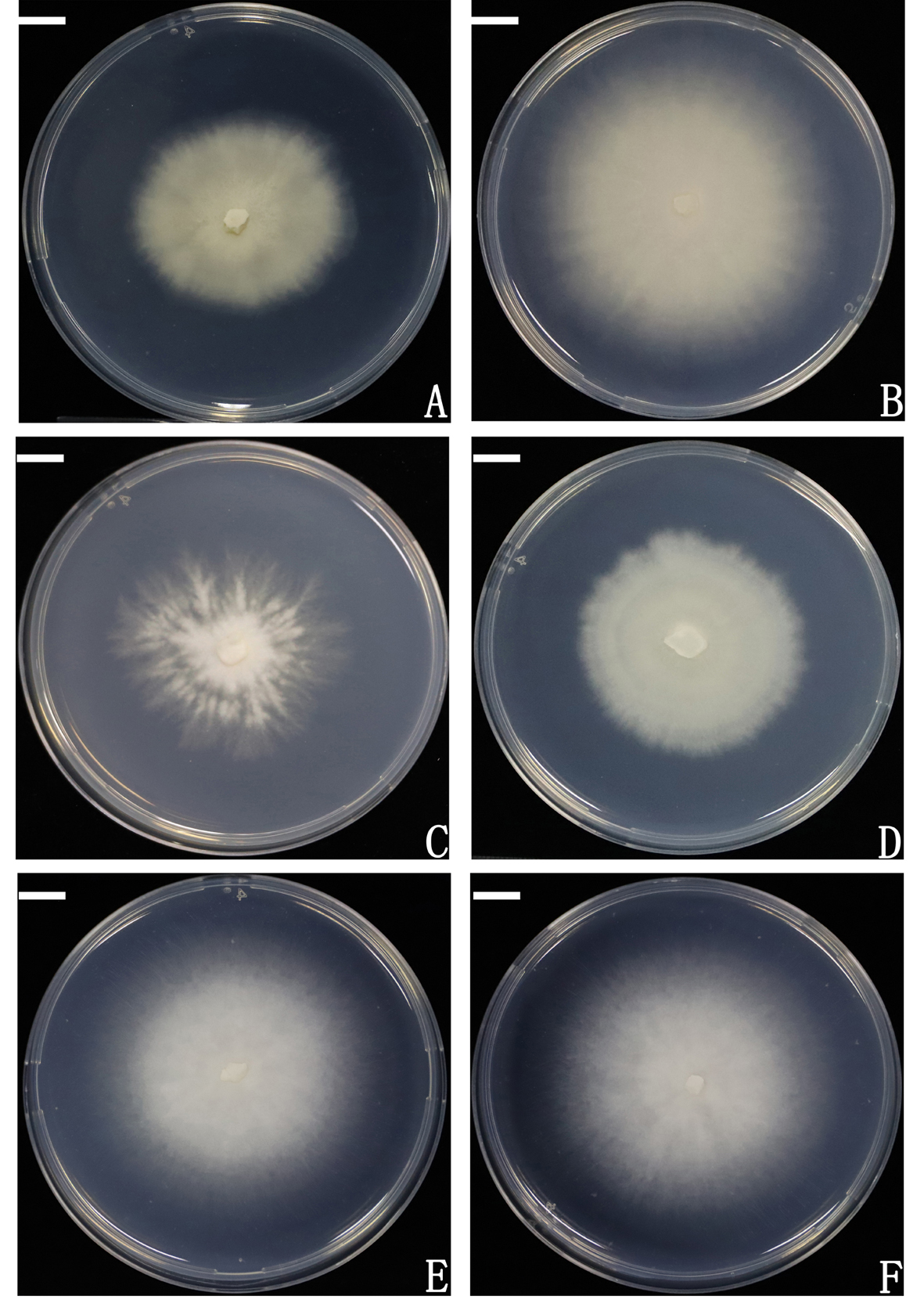

Supplement: Supplementary file 2 — Additional file 2: Figure S1. Characteristics of mycobionts at 7 days with culture on potato dextrose agar medium. (A) fungal strain S2; (B) fungal strain S3; (C) fungal strain S4; (D) fungal strain S5; (E) fungal strain S6; (F) fungal strain S7. Scale bar = 1 cm. [file 40529_2019_278_MOESM2_ESM.jpg]

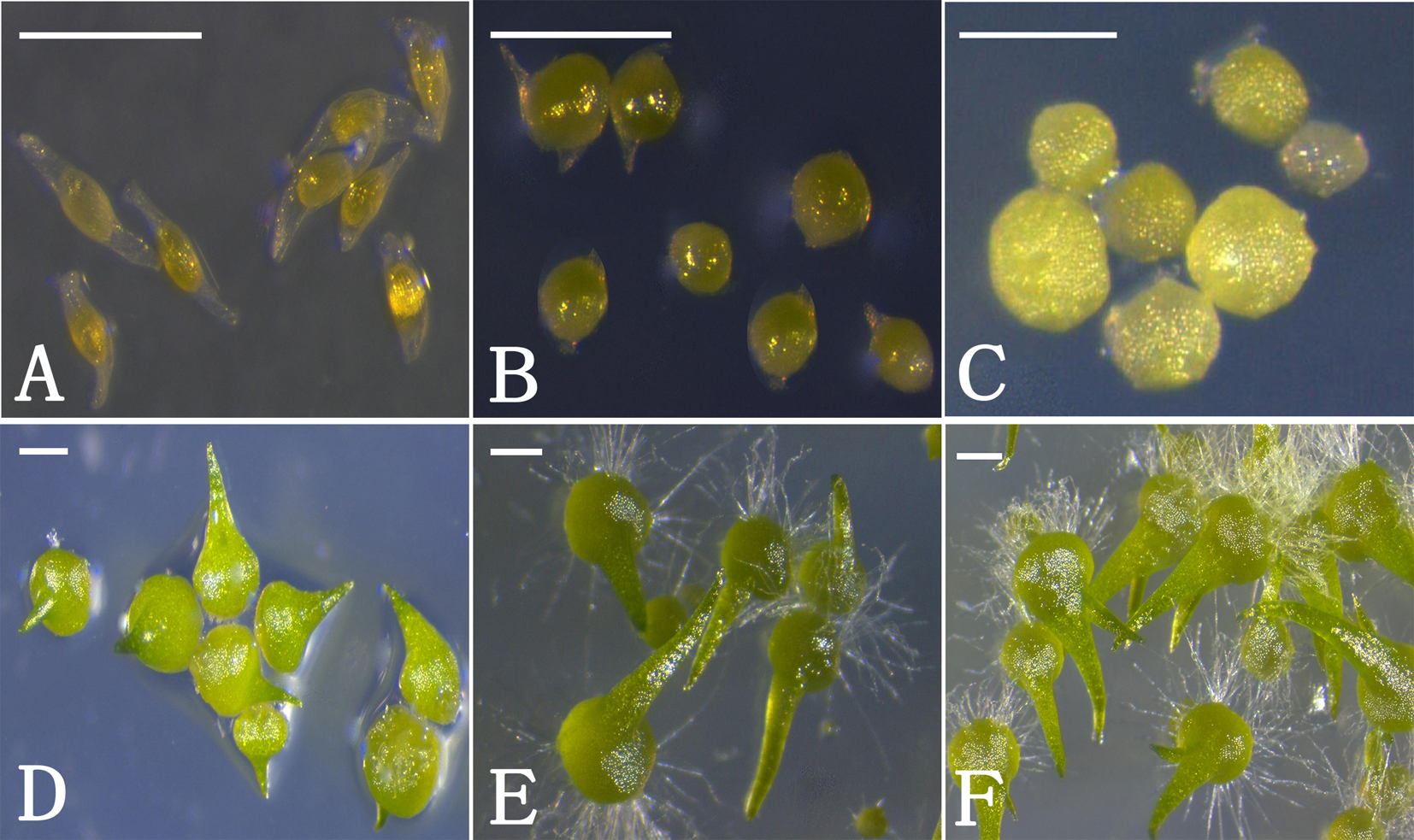

Supplement: Supplementary file 3 — Additional file 3: Figure S2. Developing stages of D. officinale from seed germination to protocorm formation. (A) Stage 0, embryos enclosed by intact seed coats. Scale bar = 0.5 mm. (B) Stage 1, swollen seeds after 1 week of inoculation. Scale bar = 0.5 mm. (C) Stage 2, swollen embryo rupturing the seed coat. Scale bar = 0.5 mm. (D) Stage3, green protocorm with shoot tip and rhizoids. Scale bar = 0.5 mm. (E) Stage 4, emergence of first leaf. Scale bar = 0.5 mm. (F) Stage 5, emergence of second leaf. Scale bar = 0.5 mm. [file 40529_2019_278_MOESM3_ESM.jpg]
